# Depression Treatment with Duloxetine and Reduction of Inability to Work

**DOI:** 10.1155/2012/264854

**Published:** 2012-08-02

**Authors:** Michael Happich, Edith Schneider, Stefan Wilhelm, Thomas Zimmermann, Alexander Schacht

**Affiliations:** ^1^Medical Department, Lilly Deutschland GmbH, 61352 Bad Homburg, Germany; ^2^Global Statistical Sciences, Lilly Deutschland GmbH, 61352 Bad Homburg, Germany

## Abstract

Data on inability to work from an observational study in patients treated with duloxetine for major depressive disorder in clinical practice in Germany were collected. Ability to work was compared between baseline and up to 6 months after initiation of duloxetine. All patients with a working status at baseline other than retired or retired early were included. 2,825 patients were analyzed, 54.8% were able to work at baseline increasing to 83.8% at 6 months. Of those patients unable to work at baseline, 72.7% were able to work after 6 months. A relevant reduction of inability to work was also found for patient subgroups with moderate to severe pain at baseline and those with and without MDD pretreatment. As inability to work is one of the main cost drivers for depressive patients in Germany, the reduction of inability to work could potentially result in considerable cost savings for health insurance companies and society.

## 1. Introduction

Depression is a major reason for disability and has recently become the most important ICD-10 diagnosis for inability to work in Germany as measured by the number of days unable to work [1]. It is also a frequent reason for hospitalization for patients in the active workforce according to the statutory health insurances in Germany [2, 3].

Several studies and reviews have shown that painful physical symptoms (PPSs) are frequent in patients with a depressive disorder [4]. In this context PPS can be defined as depression-related pain not being caused by physical handicaps (e.g., herniated disc). It has been further evaluated that comorbidity of depression and PPS results in a particular high-economic burden due to increased inability to work and hospitalizations compared to depression without comorbid PPS [5–8]. Moreover, the long-term treatment outcomes for depressive patients with comorbid PPS are worse compared to depressive patients without PPS [9, 10].

As inability to work and hospital stays are the 2 major drivers for costs in healthcare system in Germany and most healthcare systems worldwide [11], the successful and early treatment of patients with depression and comorbid PPS may potentially reduce health care costs by a considerable amount. Although treatment with any antidepressant might also reduce associated pain symptoms, the effect on pain has not been assessed for most medications. Of the modern re-uptake inhibitor medications, duloxetine has shown a direct influence on PPS in depressive patients with moderate pain at baseline in a randomized controlled clinical setting [12, 13]. In addition, duloxetine is approved for the treatment of diabetic peripheral neuropathic pain in Europe, and for fibromyalgia and chronic musculoskeletal pain in the US and other geographies [14].

To evaluate health outcomes, such as inability to work, randomized clinical trials (RCTs) are not ideally suited because only a selected population of patients and physicians participate in RCTs and can be evaluated. In addition, treatments and assessments are more intensive and tightly regulated as compared to standard clinical care. Further, RCTs in depression are usually of short duration (typically 6 to 12 weeks) and, therefore, cannot address mid- to long-term effects on working ability. To gather data that are closely matching real-life, long-term observational studies are better suited [15, 16]. Specifically, observational approaches are better suited, because prescription patterns and therapeutic decisions are at the discretion of the physician, and patient. However, unlike in well-designed RCTs results from observational studies cannot be directly interpreted as the causal relationship of the factor of interest (e.g., treatment) and the effect (e.g., inability to work). 

We are evaluating data from a large observational study in patients with major depressive disorder (MDD) treated with duloxetine in routine clinical practice in Germany. The hypothesis to be explored in this analysis is that the proportion of patients, especially the depressed patients with PPS unable to work at the start of the study, will be reduced after 6 months of duloxetine treatment. Ability to work at initiation of treatment and after 6 months of treatment is compared. In addition, we assess changes in working ability for patients with different levels of pain, pretreatment, and working ability at baseline. Thus, our results allow quantification of changes in working ability in depressive patients following treatment initiation with duloxetine.

## 2. Materials and Methods

This analysis is based on a multicenter, prospective, noninterventional study in clinical practice performed from August 2005 to December 2007 with an individual observational period of 6 months. The main objective was to investigate the influence of early changes in PPS on the long-term changes in depressive symptoms during the treatment of patients with MDD with duloxetine. The detailed description of the study design has been published by Schneider et al. [17].

The study was approved by the appropriate ethics committee. All patients provided written informed consent to the collection and release of their anonymized data.

The study was conducted at 693 office-based psychiatrists/neurologists in Germany. Data were collected at baseline (i.e., at new prescription of duloxetine), 2 weeks, 1, 3, and 6 months after baseline. Depressive symptoms were assessed by the patient using the KUSTA scale (Kurz-Skala Stimmung/Aktivierung-Short Mood/Drive Scale) [18] and by the physician-rated Inventory for Depressive Symptomatology (IDS-C) [19]. PPSs were assessed by patient-rated Visual Analogue Scales (VASs). The VAS is the most common tool used in pain-related research, as well as the standard approach to measure pain in clinical practice. Being a continuous measure, pain severity is rated ranging from 0 cm (no pain at all) to 10 cm (worst imaginable pain). A detailed description on the use of these scales and their analysis is included in Schneider et al. [17]. 

The working status of the patient and proportion of patients unable to work were collected at baseline, 3 months, and 6 months. For the present secondary analyses focusing on inability to work, all patients with a nonmissing working status other than “retired” or “retired early” at baseline were used.

All baseline descriptive variables collected in the study were analyzed for this cohort. The proportion of patients being unable to work was analyzed descriptively over time. In addition, the effectiveness scales for depression (IDS-C total score) and pain (VAS overall pain score) were also descriptively analyzed for these patients over time.

Subgroup analysis for proportion of patients unable to work, IDS-C, and VAS were performed for

- patients able to work at baseline versus not being able to work at baseline,
- patients with mild pain (≤30 mm VAS) versus moderate to severe pain (>30 mm VAS) at baseline,
- patients without pretreatment, with a single pretreatment, and with multiple pretreatments.

Data analyses were performed using SAS version 9.1.3 (or higher) statistical software (SAS Institute Inc., Cary, NC, USA). All analyses were exploratory; no confirmatory statistical tests were performed, or statements derived. 

## 3. Results

**Figure 1:**
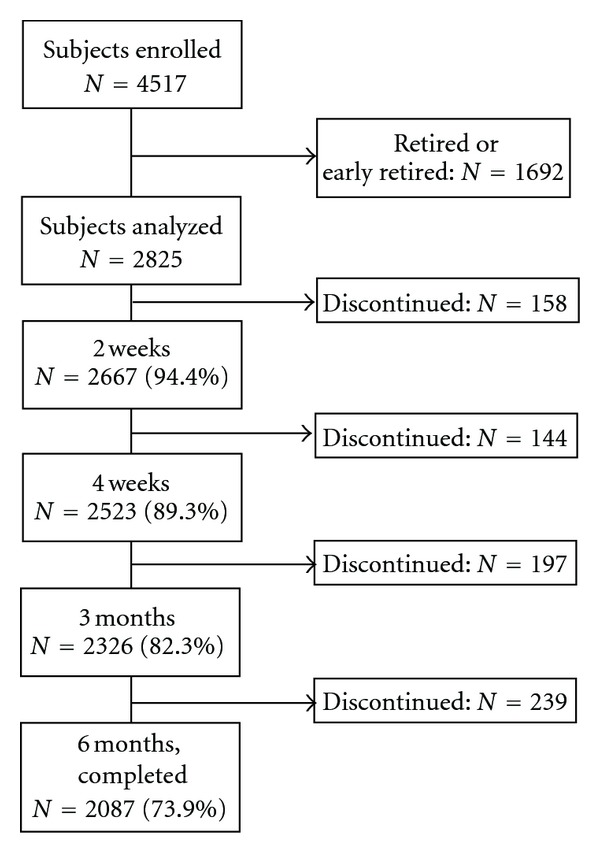
Patient disposition.

Of the 4517 patients enrolled in the study, 2,825 (62.5%) did meet the criteria for being potentially able to work and were included in the analyses.  Figure 1 shows the patient disposition of this analysis over the course of the study.

The baseline characteristics of the patients are summarized in Table 1. The majority of patients were female and 11% of the patients had been hospitalized because of depression in the preceding year. More than 70% of the patients started treatment at baseline with an initial dose of 30 mg QD duloxetine. Of all patients potentially being able to work, 1,266 (44.8%) were unable to work at the time of baseline assessment, and their baseline characteristics were analyzed separately. Most baseline characteristics of the patients unable to work at baseline were similar to those of the overall analysis population. The only differences were a higher proportion of these patients hospitalized (16.1%) and with any suicide attempt (4.3%) during the previous 12 months. Further, a longer mean duration of inability to work in the previous year (14.3 weeks) and a higher IDS-C baseline score (42.1 ± 11.87) were reported for patients unable to work at baseline.

Of all 2,825 patients in the analysis 2,185 (79.4%) had an overall pain severity VAS >30 mm at baseline and were analyzed as moderate to severe pain subgroup. Regarding pretreatment, 1,214 (43.0%) of the patients had no pretreatment for MDD at baseline, 1,219 (43.2%) had a single pretreatment, and 392 (13.9%) had combined pretreatments and were analyzed as subgroups.

**Figure 2:**
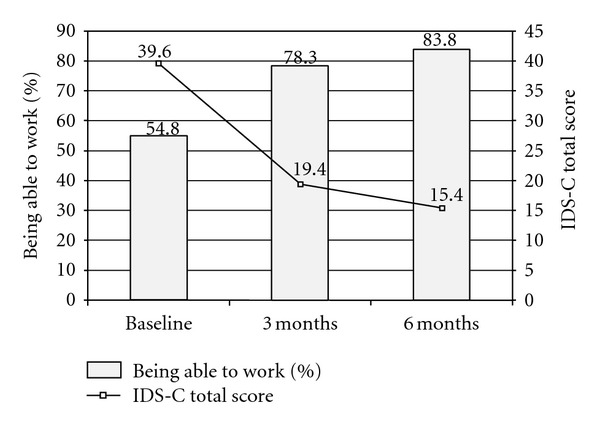
Percentage of patients being able to work and IDS-C total score at baseline, 3 months and 6 months.

The proportion of patients who were able to work at baseline, 3 months, and 6 months and the respective VAS overall pain scores and IDS-C total scores are summarized in Table 2 and Figure 2. In addition, Table 2 presents the respective results for the subgroup of patients who were unable to work at baseline. LOCF results were consistent with observed cases analysis.

**Figure 3:**
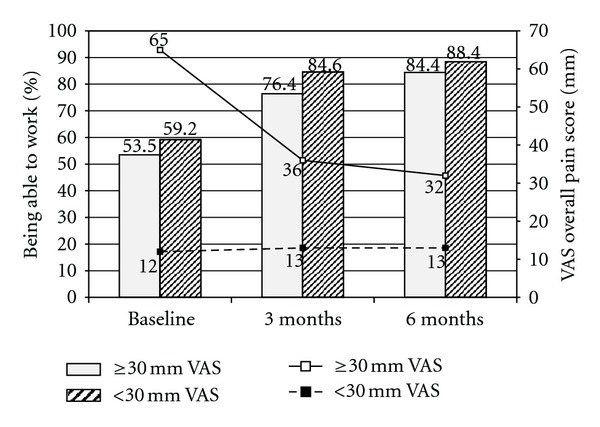
Subgroup analysis by baseline pain severity (<30 mm VAS versus ≥30 mm VAS) for % patients being able to work and mean VAS overall pain score at baseline, 3 months and 6 months.

Subgroup analyses showed a clinically relevant reduction of the proportion of patients unable to work with moderate or severe pain (Figure 3). In addition, VAS overall pain was reduced in patients with moderate or severe pain at baseline (Figure 3).

**Figure 4:**
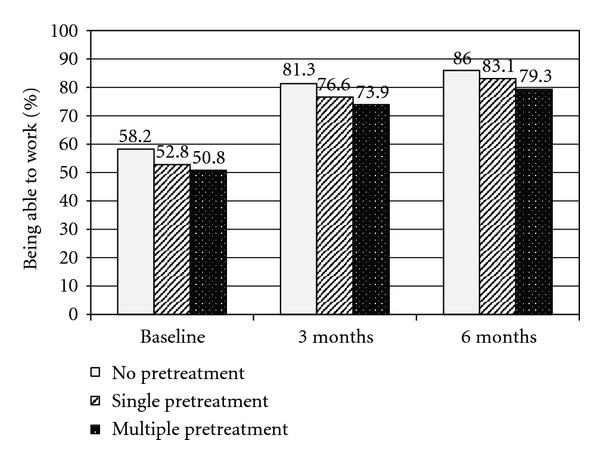
Subgroup analysis by type of pretreatment for % patients being able to work at baseline, 3 months and 6 months.

The subgroup analysis of patients by pretreatment status in the last week before start of the observation showed that the proportion of patients unable to work improved in a similar way in all subgroups that is, in patients not pretreated, in those with a single pretreatment, and in those with multiple pretreatments (Figure 4).

## 4. Discussion

Depression and the resulting inability to work have a huge impact on healthcare costs. In the German health-care system the impact of depression has been increasing in recent years, and now the ICD-10 diagnosis is resulting in the highest numbers of days unable to work in the workforce of the statutory health insurances [1]. Generally, there are few available representative databases reporting both, inability to work and medical outcomes. The present dataset includes both. 

Our results suggest that the proportion of patients unable to work is reduced after 3 and 6 months of newly started treatment with duloxetine compared to baseline at treatment initiation. Reductions of a similar magnitude were found for all subgroups analyzed: moderate to severe pain and no pretreatment versus single pretreatment versus combined pretreatment. The proportion of patients unable to work was reduced from baseline to 6 months by approximately 64%. Most notably, in the subgroup of patients unable to work at baseline 72.7% were able to work after 6 months.

Wade et al. performed an analysis of sick leave days as a comparable measure to inability to work with duloxetine and escitalopram based on the results of a 24-week RCT [20] and found that the initiation of antidepressant treatment can reduce the inability to work. However, sick leave duration for those patients are unable to work seemed to increase over the course of the study, especially for duloxetine patients. Our study reports similar findings in relation to the positive effect of antidepressant treatment in reducing inability to work. However, results are difficult to compare as Wade's results were collected in the more artificial setting of an RCT, which is not representative for clinical practice. 

The relationship between pain and depression is complicated and not fully understood, since depression may be both a cause as well as a consequence of PPS [21]. The neurobiological pathways underlying depression and pain suggest commonalities in the activity of serotonin and norepinephrine transmission [22] such that the generally high prevalence of PPS among depressed patients has led some investigators to propose that pain symptoms should be a core feature of depression [4]. Furthermore, earlier pain reduction within the first 2 to 4 weeks seems to be associated with better treatment outcomes [17], which is in line with research by Szegedi et al. [23] and Stassen et al. [24] on early improvement with antidepressant medications. As a result, it has been stated that long-term treatment outcomes of depressive patients with comorbid PPS are worse compared to depressive patients without PPS [9, 10]. With the initiation of duloxetine and its potential analgesic properties, our study could not confirm these findings after 6 months of treatment, both absolute and relative risk reductions in the inability to work were numerically better in the cohort with at least moderate pain at baseline. In the absence of a control group, however, it is difficult to attribute this outcome to duloxetine's analgesic and antidepressant efficacy.

Additionally, it should be noted that reduction in inability to work can have broader implications than purely economic ones. Romera et al. [25] found an association between earlier sick leave and subsequent level of remission, that is, that functional impairment can potentially have consequences for the treatment outcome of depression itself.

One of the main limitations of our study is that no control group without treatment initiation was available. It remains unclear how treatment with other antidepressants would have reduced the inability to work in comparison to duloxetine. Additionally, unlike in controlled clinical trials (RCTs), the results from an observational setting do not allow causal interpretation of factors of interest and effects. RCTs are the clearer approach to assess treatment differences but often lack external validity due to many selection criteria leading to a very clean population. Also, most larger RCTs are multinational, limiting their ability to include the evaluation of inability to work, as this may vary more strongly by country than by treatment. Ideally, observational studies and RCTs can complement each other. Thirdly, as is common in observational studies and long-term trials, a considerable proportion of patients dropped out of the study, which might have influenced the outcomes, for example, ability to work, as an outcome may have been overrepresented if patients, who did not respond and stayed unable to work, dropped out of the study. Indeed, discontinuation rates in our study seem slightly higher than for those of patients taking antidepressants in the European observational study by Demyttenare et al. [26]. However, in that study, the baseline patient population (with less antidepressant use and lower overall pain level) was somewhat different than in our cohort, which might explain differences in discontinuation. Despite this, the discontinuation rate for our study was in the range seen for antidepressants in clinical trials [27], and last observation carried forward (LOCF) results were generally consistent with the observed cases suggesting that the impact of drop out is limited in our study. Finally, the data were gathered in Germany only. Depending on the type of healthcare system, costs related to inability to work impact health care expenses in different ways.

## 5. Conclusions

During treatment of depressive patients with duloxetine, a considerable reduction of inability to work after 3 and 6 months of treatment, overall, and in various subgroups was observed. As inability to work is one of the main cost drivers for depressive patients in Germany, the reduction of inability to work could potentially contribute to considerable cost-savings for health insurance companies and society.

## Figures and Tables

**Table 1:** Baseline characteristics.

Variable	*n* (%)	Mean (SD)
Age (years; *N* = 2822)		46.4 (10.0)
Gender: female (*N* = 2824)	2050 (72.6)	
Currently unable to work^ a^ (*N* = 2825)	1266 (44.8)	
Duration of inability to work in the last 12 months (weeks; *N* = 2732)		7.8 (13.5)
Age at onset of depression (years; *N* = 2779)		37.2 (11.7)
Time since onset of depression (years; *N* = 2777)		9.2 (9.4)
Any hospitalization during the last 12 months (*N* = 2802)	316 (11.2)	
Any suicide attempt during the last 12 months (*N* = 2793)	74 (2.6)	
Duloxetine starting dose (*N* = 2816)		
30 mg	2051 (72.6)	
60 mg	756 (26.8)	
Reason to start or change to duloxetine (*N* = 2825)		
Inadequate efficacy of pretreatment	1365 (48.3)	
Initial treatment	1350 (47.8)	
Patient decision	412 (14.6)	
Inadequate tolerability of pretreatment	314 (11.1)	
History of antidepressant therapy in the last week (*N* = 2825) yes	1611 (57.0)	
No antidepressant therapy	1214 (43.0)	
Single antidepressant treatment	1219 (43.2)	
Most common (>2% of patients) single antidepressant therapies:		
Tricyclic antidepressant	515 (18.2)	
Selective serotonin reuptake inhibitor	410 (14.5)	
Noradrenergic and specific serotonergic antidepressant	126 (4.5)	
Selective serotonin and noradrenaline reuptake inhibitor	74 (2.6)	
Multiple antidepressant treatment	392 (13.9)	
Patients with overall pain VAS >30 mm	2185 (79.4)	
Any permanent pain medication (*N* = 2813)	769 (27.2)	
Any on demand pain medication in the last 12 months (*N* = 2801)	1744 (61.7)	
Any concomitant somatic diseases (*N* = 2825)	1813 (64.2)	
Most common (>9% of patients) concomitant somatic diseases:^b^		
Muscle and skeleton diseases	871 (30.8)	
Hypertension	573 (20.3)	
Allergies	306 (10.8)	
Metabolic diseases	277 (9.8)	
Gastrointestinal diseases	275 (9.7)	
Neurologic diseases	261 (9.2)	

*N*: number of patients with available data; *n*: number of patients in category; SD: standard deviation.

^a^Answer “yes” to the question “Is the patient unable to work today?”.

^b^As selected from the check list.

**Table 2:** Working ability, depression, and pain scores over time: all patients and patients unable to work at baseline.

Variable	All patients (*N* = 2825)		Unable to work at baseline (*N* = 1266)	
	*n* (%)	95% CI	*n* (%)	95% CI
Patients being able to work				
Baseline	1536 (54.8)	53.0; 56.7	—	—
3 months	1755 (78.2)	76.4; 79.9	622 (62.0)	58.9; 65.0
6 months	1611 (83.8)	82.1; 85.4	610 (72.7)	69.6; 75.7
LOCF	1859 (81.3)	79.7; 82.9	707 (69.2)	66.2; 72.0
IDS-C total score	mean	SD	mean	SD
Baseline	39.6	12.2	42.1	11.9
3 months	19.4	12.8	20.9	13.1
6 months	15.4	12.2	17.2	13.1
LOCF	18.0	13.6	19.9	14.5
VAS overall pain score [mm]	mean	SD	mean	SD
Baseline	53.9	26.8	56.5	26.9
3 months	31.7	25.4	33.3	25.6
6 months	28.1	25.2	30.4	26.2
LOCF	30.8	26.4	33.3	27.4

CI: confidence interval; IDS: inventory of depressive symptomatology; LOCF: last observation carried forward; *N*: number of patients in category (percentages are based on the actual number of patients with nonmissing information at the respective visit); SD: standard deviation; VAS: visual analog scale.
